# Supplementary material for: Automated cell boundary and 3D nuclear segmentation of cells in suspension
Source: Sci Rep. 2019 Jul 15;9:10237. doi: 10.1038/s41598-019-46689-5 (PMC6629630; doi:10.1038/s41598-019-46689-5)
Supplement: Supplementary file 1 — Supplementary Material [file 41598_2019_46689_MOESM1_ESM.docx]

**SUPPLEMENTARY MATERIAL**

**Automated cell boundary and 3D nuclear segmentation of cells in suspension**

Benjamin Kesler, Guoliang Li, Alexander Thiemicke, Rohit Venkat and Gregor Neuert

correspondence to: gregor.neuert@vanderbilt.edu

**Figure S1. Segmentation of Live *S. Cerevisiae***. Live S. Cerevisiae cells with a nuclear RFP marker (NRD1) were imaged. Displayed is an overlay of widefield (grey), RFP (blue), nuclear (yellow) and cytoplasmic (magenta) segmentation by CellDissect.

**Figure S2. Comparison of Nuclear and Cellular Characteristics of Adherent versus Trypsinized mESC Cells**. Histograms (above) and bar plots (below) are shown comparing the nuclear volume, nuclear area, integrated nuclear intensity, and cell area between n = 231 adherent cells and n= 487 trypsinized mouse embryonic stem cells from the same biological replica. For the bar graph, each value is the mean normalized to adherent cells and the error bars are the standard deviations of the distributions above.

**Figure S3. Histograms of Nuclear and Cellular Aspects**. Histograms of nuclear volume (left), nuclear area (middle), and cell area (right) are shown. Each row is a different cell type, with n as the total number of cells combining all replicas. Replicas 1, 2, and 3 are blue, red, and yellow respectively, and overlapping bar colors are determined by subtractive color mixing (e.g. blue and yellow combine to show green). If all bars are overlapping, the color is golden.

**Figure S4. Bar Plots of Nuclear and Cellular Aspects**. Bar plots of the natural logarithm of nuclear volume, nuclear area, and cell area are shown. Bars are grouped either by cellular aspect quantified (top) or cell type (bottom). Error bars are standard deviations of the mean between N = 3 biological replicas.
